# PD-1^+^ CD4 T cell immune response is mediated by HIF-1α/NFATc1 pathway after *P. yoelii* infection

**DOI:** 10.3389/fimmu.2022.942862

**Published:** 2022-08-24

**Authors:** Haixia Wei, Anqi Xie, Jiajie Li, Chao Fang, Lin Liu, Junmin Xing, Feihu Shi, Feng Mo, Dianhui Chen, Hongyan Xie, Quan Yang, Xingfei Pan, Xiaoping Tang, Jun Huang

**Affiliations:** ^1^ Department of Infectious Diseases, Guangdong Provincial Key Laboratory of Major Obstetric Diseases, The Third Affiliated Hospital of Guangzhou Medical University, Guangzhou, China; ^2^ Department of Basic Medical Science, China Sino-French Hoffmann Institute, Guangzhou Medical University, Guangzhou, China; ^3^ Guangzhou Eighth People’s Hospital, Guangzhou Medical University, Guangzhou, China; ^4^ Guangzhou Municipal and Guangdong Provincial Key Laboratory of Protein Modification and Degradation, School of Basic Medical Sciences, Guangzhou Medical University, Guangzhou, China

**Keywords:** CD4 T cells, PD-1, *P. yoelii*, NFATc1, HIF-1α

## Abstract

The morbidity and mortality of malaria are still high. Programmed cell death-1(PD-1) is an important co-inhibitory factor and CD8 T cells with PD-1 were reported to be exhausted cells. It remains unknown what the role of CD4 T cells expressing PD-1 is and what the upstream regulating molecules of PD-1 in CD4 T cells are. The C57BL/6 mice were injected with *Plasmodium yoelii* (*P. yoelii*) in this study. Expressions of PD-1, activation markers, and cytokines were tested. The differentially expressed genes between PD-1^+/-^ CD4 T cells were detected by microarray sequencing. Western blot, chromatin immunoprecipitation (ChIP), siRNA, hypoxia inducible factor-1α (HIF-1α) inducer and inhibitor were used to explore PD-1’s upstream molecules, respectively. The proportions of PD-1^+^ CD4 T cells increased post *P. yoelii* infection. PD-1^+^ CD4 T cells expressed more activated surface markers and could produce more cytokines. Nuclear factor of activated T cells 1 (NFATc1) was found to be a key transcription factor to induce PD-1 expression after infection. Both the inducer and the inhibitor of HIF-1α could change the expressions of NFATc1 and PD-1 *in vivo* and *in vitro*, respectively. Taken together, *P. yoelii* infection induced NFATc1 expression by HIF-1α. The highly expressed NFATc1 entered the nucleus and initiated PD-1 expression. PD-1^+^ CD4 T cells appeared to be more activated and could secrete more cytokines to regulate the host’s immune responses against malaria.

## Introduction

Malaria seriously threatens human health in the world. Malaria, acquired immune deficiency syndrome (AIDS), and tuberculosis are considered to be the three most serious infectious diseases in the world (1). In 2020, it is estimated that there are 241 million cases of malaria worldwide (2). Malaria mainly causes anemia and splenomegaly. Unfortunately, there are still many difficulties in the prevention and treatment of malaria.

T cells are very important in the host against *Plasmodium* immunity (3). CD4 T cells are the centrality to engineering essential elements of host immunity in the course of *Plasmodium* infection (3, 4). CD4 T cells can be activated following the engagement of antigens presented by MHC class II molecules. Then they differentiate into different subsets. Helper T 1 (Th1) cells are the major cells secreting tumor necrosis factor-α (TNF-α) and Interferon-γ (IFN-γ) in the mice infected with *Plasmodium* (5). IFN- γ and TNF- α are essential for the protective immune response of the host against *Plasmodium* (6–8), which could synergistically induce a series of following responses to control the load of parasites in the host (4, 9, 10). Furthermore, T follicular helper (Tfh) cells could facilitate B cells to produce functional anti-parasitic antibodies (4).

It was reported that CD8 T cells expressing programmed cell death-1(PD-1) will be exhausted, and PD-1 worked as an inhibitory molecular in the process of CD8 T cell differentiation (11). The exhaustion of CD8 cells has different stages. Stage I, TNF- α and Interleukin-2 (IL-2) produced by CD8 cells and the cytotoxicity reduce gradually. Stage II, CD8 T cells with PD-1 fail to secreting IFN-γ, IL-2, or TNF-α with antigenic stimulation. Stage III, all effector functions are thoroughly disappearing (12). In the rodent malaria model, PD-1 leads to more than 95% reduction in numbers and functions of *Plasmodium*-specific CD8 T cells, which could result in chronic infection (13). In some patients with acute febrile malaria, the percentage of PD-1 expressing NK cells increased, which contributed to the diminished natural cytotoxicity and enhanced antibody-dependent cellular cytotoxicity (14). The function of CD4 T cells expressing PD-1 is relatively complex and is not thoroughly uncovered. Some scholars believed that CD4 T cells expressing PD-1 would also be exhausted similar to CD8 T cells (15, 16). Others found that PD-1 was a specific surface molecule of Tfh cells. PD-1^+^ Tfh cells were not exhausted T cells but worked as the core of maintaining antigen-specific effects (17–19). One aim of the this study was to explore the role of PD-1 expressing CD4 T cells in in *P. yoelii* infected C57BL/6 mice.

It was reported that PD-1 expression was regulated by 8 transcriptional activators (NFATc1, STAT3, Notch, FoxO1, c-fos/AP-1, ISGF3, STAT4, NF- κB) and 2 transcriptional repressors (T-bet, Blimp-1) (20). Hypoxia inducible factor-1α (HIF-1α), a transcriptional factor, could be degraded by ubiquitinated proteasome pathway after translation under a normoxic environment. However, in a hypoxic environment, HIF-1α could not be degraded by the ubiquitination pathway. HIF-1α could transfer into the cell nucleus, regulate the expressions of downstream genes, and then initiate many kinds of life functions, including hypoxia adaptation, inflammatory response, tumor growth, etc (21). Anemia is one of the common symptoms of acute malaria. It is well known that red blood cells play important roles in transporting oxygen. So it would be a hypoxic environment for the body, and many molecules would be regulated in acute malaria. PD-1 ligand 1 (PD-L1) was reported to be a novel direct target of HIF-1α (22). In tumor model, alleviating tumor hypoxia could improve the efficacy of PD-1 antibody (23). However, there is no evidence to show whether HIF-1α is upstream molecule of PD-1.

This study tried to research the phenotypes and functions of PD-1^+^ CD4 T cells in *P. yoelii* infected C57BL/6 mice. Furthermore, the upstream regulating molecules of PD-1 in CD4 T cells were also explored. Our results might be helpful to develop a novel immune-based treatment for malaria in the future.

## Materials and methods

### Mice

Wild-type female C57BL/6 mice (6–8 weeks) were purchased from Guangzhou University of Chinese Medicine and maintained with specific pathogen-free conditions. The animal study was approved by the Institutional Animal Care and Use Committee of Guangzhou Medical University (S2020-055).

### Parasites

*Plasmodium yoelii nigeriensis* NSM was preserved in our lab. The preserved parasites were thawed in the mice until the parasitemia was about 15%. The naive mice were intraperitoneally injected with 1 × 10^6^ infected red blood cells (iRBCs).

### Isolation of splenic lymphocytes

12 days after infection, spleens were surgically removed and weighed. They were mechanically ground and filtered with 100-μm cell strainers (BD Falcon, United States). Then the erythrocytes were removed by RBC lysis buffer (Pythonbio, China), and the remaining cells were washed with Hanks’ buffer and resuspended in RPMI-1640 medium.

### Reagents and antibodies

Lificiguat (YC-1) was obtained from Selleck (United States) (Cat: S7958). CoCl_2_ · 6H_2_O was purchased from Merck (Germany) (Cat: C8661). The following fluorescence-labeled antibodies were purchased from BD (United States), R&D (United States), or Biolegend (United States): Percp-cy5.5-CD4 (GK1.5), APC-PD-1 (29F.1A12), FITC-CD3 (145-2C11), Percp-cy5.5-CD19 (6D6), APC-cy7-CD8 (54-6.7), PE-cy7-CD11b(M1/70), APC-CD62L (MEL-14), PE-CD25(BC96), PE-CD40L (MR1), APC-CD69 (H12F3), APC-IL-17(TC11-18H10.1), BV421-CXCR5 (L138D7), APC-IFN-γ (XMG1.2), PE-IL10 (JES5-16E3), PE-IL4 (11B11), PE-IL2 (JES6-5H4), anti-mouse CD16/CD32 (2.4G2), Alexa Fluor 488-NFATc1(7A6), APC- HIF-1α(241812). Anti-NFATc1 rabbit mAb (D15F1), anti-tubulin antibody (Cat: 2144S), anti-histone H3 rabbit mAb (D1H2), and HRP-labled secondary antibodies (Cat: L3012-2) were purchased from cell signaling technology (CST, United States). The eFluor506-FVD (Cat: 65-0863-14) was purchased from eBioscience (United States).

### Detection of peripheral red blood cells

The peripheral blood from mice was collected in EDTA pretreated tubes and then examined in the Clinical Laboratory Department of the Sixth People’s Hospital of Panyu (Guangzhou, China).

### Flow cytometry (FCM) analysis

For cell surface marker detection, single lymphocytes were stained with fluorescence-labeled anti-PD-1, CD11b, CD3, CD19, CD4, CD8, CD62L, CD69, CD40L, CD25, and CXCR5 antibodies at 4°C for 30 min. For cytokine detection, single lymphocytes were stimulated with phorbol 12-myristate 13-acetate (PMA, 20 ng/ml, Sigma, Germany) and ionomycin (1 μg/ml, Sigma) at 37°C for 1 h, then brefeldin A (10 μg/ml, Sigma, Germany) was used to stop the stimulation. Cells were incubated with antibodies for cell surface markers firstly, then they were fixed with 4% paraformaldehyde, and permeabilized. The cells were incubated with fluorescence-labeled antibodies for IFN-γ, IL-10, IL-17, IL-4, and IL-2 for 30 min. To detect the expression of NFATc1 and HIF-1α, cells were treated with a transcription factor staining kit (00-5523-00, Invitrogen,United States), and incubated with fluorescent antibodies for NFATc1 and HIF-1α. Stained cells were determined by FCM and the results were analyzed by CytExpert 1.1 (Beckman coulter, United States).

### Cell sorting

PD-1^+^ and PD-1^−^ CD4 T cells were selected by FCM sorting. Briefly, lymphocytes were incubated with fluorescein-labeled antibodies for CD4 and PD-1. Then, the positive cells were sorted by cell sorting machine (BD, Mountain View, CA, United States). CD4 T cells were sorted by magnetic beads (130-042-102, MiltenyiBio, Germany). Briefly, splenic lymphocytes were incubated with the magnetic beads conjugated anti-CD4 antibodies at 4°C for 30 min. The incubated cells were transferred to the rinsed columns and washed in the magnetic field. Finally, the magnet was removed, and the target cells were washed from the columns. The purity of the selected cells was determined by FCM analysis.

### Western blot

The nuclear and cytoplasmic proteins from PD-1^+^ and PD-1^-^ CD4 T cells were extracted by Nuclear Protein Extraction Kit (P0027, Beyotime, China). The extracted proteins were separated by electrophoresis and then transferred to PVDF membrane. Then, the PVDF membranes were blocked, incubated in the primary and HRP-conjugated secondary antibodies. The membranes were captured by the ChemiDocTM Touch Imaging System (Bio-Rad, United States).

### Chromatin immunoprecipitation (ChIP)

The splenic lymphocytes from normal and *P. yoelii* -infected mice were cross-linked with 1% formaldehyde. Then, the cells were treated with truChIP Chromatin Shearing Kit (PN 520127, Covaris,United States) for chromatin shearing. The efficiency of chromatin shearing was determined by DNA agarose electrophoresis. SimpleChIP Plus Enzymatic Chromatin IP Kit (Magnetic Beads)(CST, 9005) and antibodies were then used to pull down the binding DNA sequences. The results were determined by quantitative PCR (qPCR).

### siRNA and qPCR

siRNAs were purchased from Ribo-bio Company (Guangzhou, China) and used at 100 nM working concentration. The sorted CD4 T cells were transfected with siRNAs. In parallel, the control siRNA was also transfected to the selected cells. To detect the expression of the genes, cells were cryopreserved with TRIzol (Invitrogen, United States). Then, RNA was extracted. qPCR was performed with a CFX96 System (Bio-Rad, United States). The detail information for the used siRNAs and primers was provided in **Table 1**.

**Table 1:** siRNA and primer sequences list.

Name	sequences
NFATc1 siRNA	5’-CCGTCACATTCTGGTCCAT-3’
NFATc1 qPCR primer	F:5’-AGCTGTTCCTTCAGCCAATC-3’; R:5’- TGCCAGAGGACAGGAAGTAT-3’
PD-1 qPCR primer	F:5’-CCCTAGTGGGTATCCCTGTATT-3’; R:5’- TCCTTCAGAGTGTCGTCCTT-3’
PD-1 promoter primer	F:5’-CCTCACCTCCTGCTTGTCTCTC-3’; R:5’- GTGAGACCCACACATCTCATTGC-3’
HIF-1α qPCR primer	F:5’-CCCATTCCTCATCCGTCAAATA-3’; R:5’-GGCTCATAACCCATCAACTCA-3’
Actin qPCR primer	F:5’-GCCTTCCTTCTTGGGTATGGAA-3’; R:5’-CAGCTCAGTAACAGTCCGCC-3’

### Microarray analysis

PD-1^+^ and PD-1^−^ CD4 T cells from the spleens of *P. yoelii* infected mice were isolated by FCM sorting. The sorted cells were lysed in TRIzol and sent to Capitalbio Technology Corporation (Beijing, China) for microarray gene expression detection. Differentially expressed genes were identified significantly with fold change (FC) ≥ 2 or FC ≤ 0.5 and *p* < 0.05.

### Statistics

Statistical analyses were mainly performed in Prism using a two-tailed Student’s t-test. The differences for multiple comparisons were analyzed by one-way ANOVA with the SPSS software package (version 20). The difference was considered significant with *p* < 0.05.

## Results

### More CD4 T cells expressed PD-1 in mice with *P. yoelii* infection

**Figure 1:**
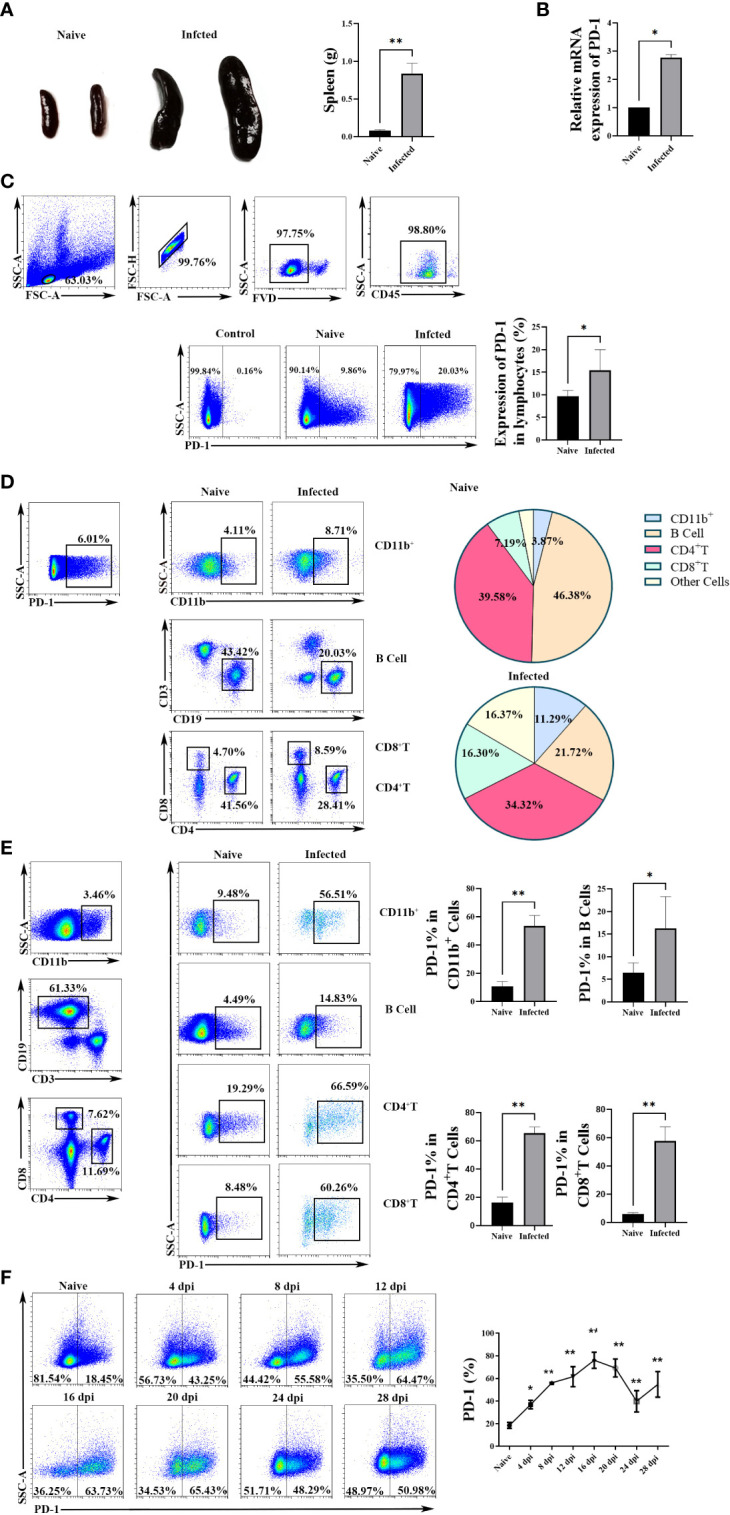
The expression of PD-1 in CD4 T cells increased upon *P. yoelii* infection. **(A)** the representative spleens from the naive and infected mice and the comparison of splenic weight from the naive and infected mice. PD-1 expression in splenic lymphocytes was tested by qPCR **(B)** and FCM **(C)**. **(D)** the constitutes of splenic PD-1^+^ cells and their changes after *P. yoelii* infection were explored by FCM. **(E)** the proportions of PD-1^+^ CD4 T cells, PD-1^+^ B cells, PD-1^+^ CD11b^+^ cells, and PD-1^+^ CD8 cells from naive and infected mice were compared. **(F)** the dynamic changes of splenic PD-1^+^ CD4 T cells in *P. yoelii* infected mice were investigated from 0-28 days, and the naive mice were set as control (0 days). 3–7 samples were prepared for each group, and the experiments were repeated three times. The error bars represented number of mice. *p < 0.05, **p < 0.01.

The mice were injected with *P. yoelii* intraperitoneally. Twelve days later, spleens were removed. The spleens of the infected mice significantly became bigger and heavier (*p *< 0.01) (**Figure 1A**). The expression of PD-1 in splenic lymphocyte suspensions from the infected mice was higher than that of the naive mice (*p* < 0.05, **Figures 1B, C**).

Subsequently, the constitutions of splenic PD-1^+^ cells were examined before and after *P. yoelii* infection. As shown in **Figure 1D**, 39.58% of the PD-1^+^ cells were CD4 T cells in the naive mice, and CD4 T cells were the major cells to express PD-1 after *P. yoelii* infection (34.32%). The percentages of PD-1^+^ B cells significantly reduced after infection (from 46.38% to 21.72%). While, the proportions of CD8^+^ cells, CD11b^+^ cells, and other cells in PD-1^+^ cells remarkably increased after *P. yoelii* infection.

Furtherly, as shown in **Figure 1E**, compared with the naive group, the proportions of PD-1^+^ CD4 T cells, PD-1^+^ B cells, PD-1^+^ CD11b^+^ cells, and PD-1^+^ CD8^+^ cells increased significantly in *P. yoelii* -infected mice (*p* < 0.01).

Then, the dynamic changes of PD-1^+^ CD4 T cells were investigated from 0-28 days of the *P. yoelii* -infected mice. The percentages remarkably increased with the prolongation of infection and picked on day 16 (about 80%), and then the percentages gradually decreased (**Figure 1F**). These results suggested that PD-1 molecule of CD4 T cells in the spleen could participate in the process of host against *P. yoelii* infection.

### The phenotype and cytokine expression profile of PD-1^+^ CD4 T cells were changed upon *P. yoelii* infection

**Figure 2:**
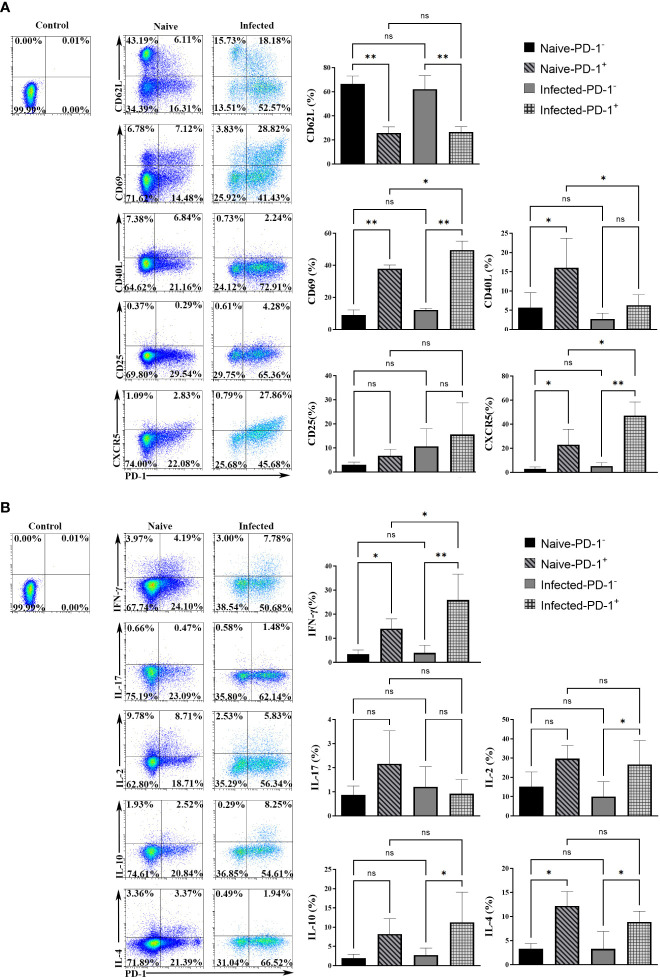
The surface markers and cytokines in PD-1^+^ CD4 T cells were changed upon *P. yoelii* infection. **(A)** single splenic lymphocytes were stained with fluorescent antibodies: PD-1, CD4, CD62L, CD69, CD40L, CD25, and CXCR5. The isotype controls were set. The expression of surface markers from different groups was compared. **(B)** the single splenic lymphocytes incubated with fluorescent antibodies: CD4, PD-1, IL-2, IFN-γ, IL-10, IL-17, IL-4. The isotype controls were set. The secreting ability of cytokines from different groups was compared. 3–7 samples were prepared for each group, and the experiments were repeated three times. The error bars represented number of mice. *p < 0.05, **p < 0.01. ns, no statistical significance.

To study the phenotypic changes of PD-1^+^ CD4 T cells post-infection, single splenic lymphocytes were stained with fluorescent antibodies for surface markers: CD4, PD-1, CD62L, CD69, CD40L, CD25, and CXCR5, respectively. The isotype controls were set. Results (**Figure 2A**) showed that more PD-1^+^ CD4 T cells expressed CD69 and CXCR5 (*p* < 0.01), and fewer of them expressed CD62L when comparing with PD-1^-^ CD4 T cells (*p* < 0.01). The expression of CD25 among the four groups was similar (*p* > 0.05). In addition, compared with PD-1^+^ CD4 T cells from naive mice, less of this kind of cells expressed CD40L, and more of them expressed CD69 and CXCR5 in the infected mice (*p* < 0.01). The above results illustrated that PD-1^+^ CD4 T cells appeared to be activated.

Cytokines expressed by PD-1^+^ CD4 T cells were examined. The single splenic lymphocytes from the naive and infected mice were stimulated with PMA and ionomycin. Then the stimulated lymphocytes were stained with fluorescent antibodies: CD4, PD-1, IFN-γ, IL-17, IL-2, IL-10, and IL-4. As shown in **Figure 2B**, in the naive mice, the percentages of PD-1^+^ CD4 T cells secreting IFN-γ and IL-4 were higher than that of PD-1^-^ CD4 T cells. While, in the infected mice, the percentages of PD-1^+^ CD4 T cells that expressed IFN-γ, IL-10, IL-2, and IL-4 were significantly higher when compared with that of PD-1^-^ CD4 T cells (*p* < 0.05). The differences in IL-17 expression among the four groups were not significant (*p* > 0.05). In addition, compared with PD-1^+^ CD4 T cells of the naive mice, more PD-1^+^ CD4 T cells of the infected mice secreted IFN-γ (*p* < 0.01). The above results illustrated that PD-1^+^ CD4 T cells could secrete more cytokines to regulate the immune response of host against malaria infection.

### There were some differences in gene expressions between PD-1^+/-^ CD4 T cells

**Figure 3:**
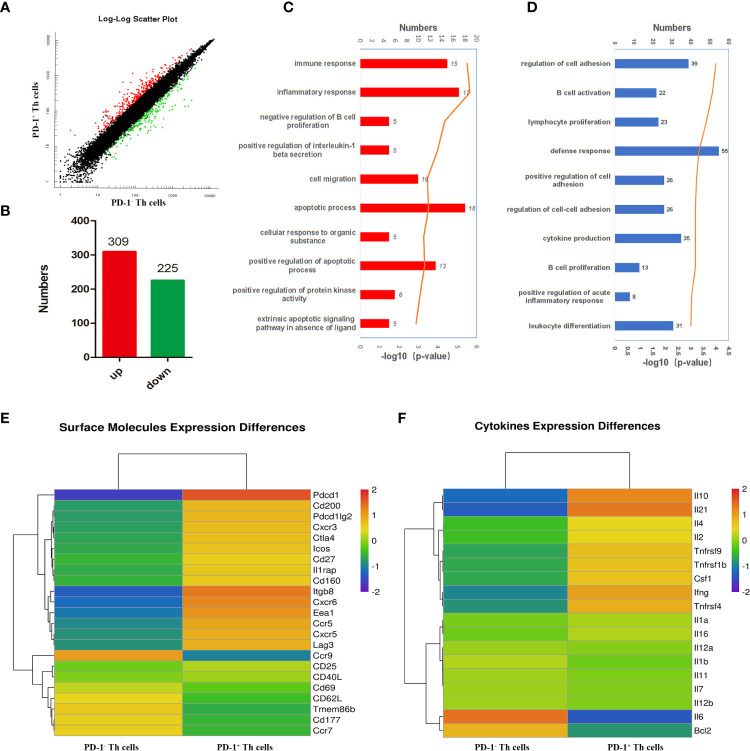
Bioinformatics analysis of the differently expressed genes between PD-1^+/-^ CD4 T cells post *P. yoelii* infection. The PD-1^+^ and PD-1^-^ CD4 T cells from the spleens of mice infected with *P. yoelii* were sorted and sequenced by microarray transcriptome. **(A)** the scatter plot showing the comparison of signal values from PD-1^+^ CD4 T cells and PD-1^-^ CD4 T cells, the dots marked in red were the up-regulated genes, the dots marked green were down-regulated genes, and the dots marked black were similarly expressed genes. **(B)** numbers of up-regulated and down-regulated genes in CD4^+^ PD-1^+^ T cells. **(C)** GO analysis showed these differentially expressed genes are mainly participated in immune response, inflammatory response. **(D)** KEGG analysis showed these genes are mainly involved in the proliferation and differentiation of lymphocytes, especially B cells. **(E)** The heatmap for the surface molecules expressed in PD-1^−/+^ CD4 T cells. **(F)** The heatmap for the cytokines expressed in PD-1^−/+^ CD4 T cells.

To further comprehensively uncover the function of CD4 T cells with PD-1 and the possible upstream regulating genes of PD-1, microarray transcriptomic sequencing for PD-1^+/-^ CD4 T cells was performed. The sequencing data of the transcriptome were shown in **Table S1**. There were 309 up-regulated and 225 down-regulated genes in PD-1^+^ CD4 T cells (**Figures 3A, B**). To obtain a comprehensive view of the screened 534 genes, Gene Ontology (GO) and Kyoto Encyclopedia of Genes and Genomes (KEGG) analyses were performed to identify the significantly enriched functional terms. The results of GO analysis revealed that these differentially expressed genes were mainly involved in “immune response”, “inflammatory response”, et al. (**Figure 3C**). KEGG analysis results revealed that the selected genes were mainly involved in “B cell activation”, “lymphocyte proliferation”, “cytokine production”, “B cell proliferation”, “leukocyte differentiation”, et al. (**Figure 3D**). In addition, the analysis data of transcriptome further confirmed that PD-1^+^ CD4 T cells expressed more surface-activated molecules such as CXCR3, CXCR5, ICOS, etc (**Figure 3E**). The expressions of cytokine from the two groups were also explored, and PD-1^+^ CD4 T cells expressed more IL-10, IL-21, IL-4, IL-2, and IFN-γ, etc (**Figure 3F**). The sequencing results further implied that PD-1^+^ CD4 T cells could be more activated and could secrete more cytokines to modulate against malaria immunity.

### PD-1 expression was induced through NFATc1 post *P. yoelii* infection

**Figure 4:**
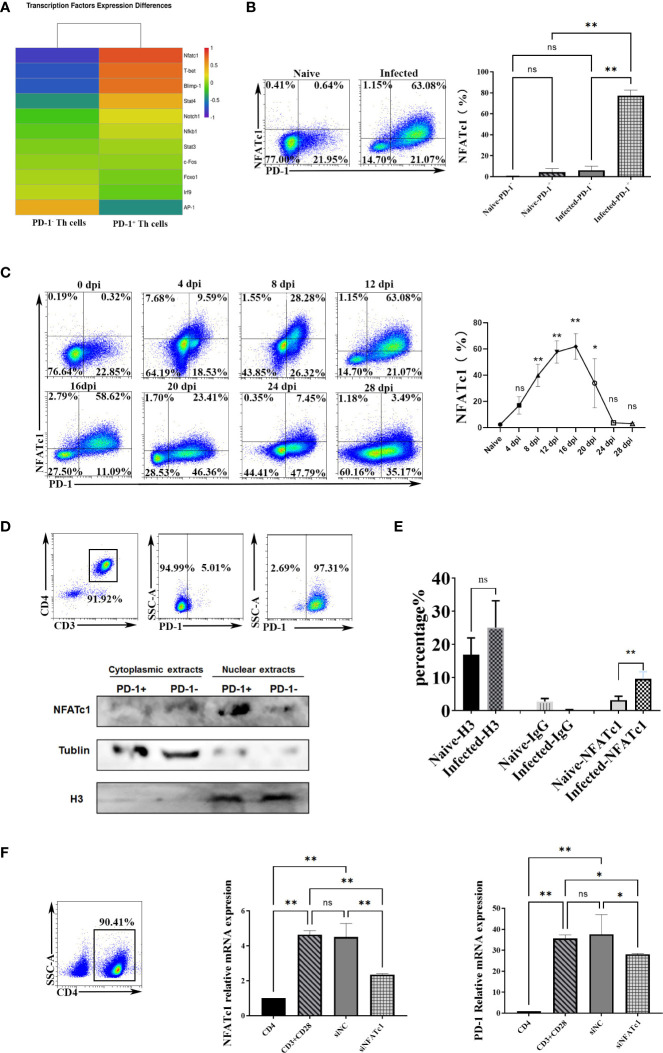
*P. yoelii* infection induced PD-1 expression through NFATc1. **(A)** the expression of the reported 10 transcriptional activators/repressors from the sequencing data. **(B)** the expressions of NFATc1 in PD-1^+/-^ CD4 T cells were examined by FCM. **(C)** the dynamic changes of NFATc1 expression in CD4 T cells of *P. yoelii* -infected mice were monitored from 0-28 days. **(D)** the PD-1^+^ and PD-1^-^ CD4 T cells from the spleen of mice infected with *P. yoelii* were sorted. The cytoplasmic and nuclear proteins were extracted, and the expression of NFATc1 was detected by Western. **(E)** in the ChIP experiment, anti-histone H3 antibody, and normal IgG were set as controls. Anti- NFATc1 antibody was used to pull down the binding DNA sequences. Then, qPCR was used to detect the binding efficiency. **(F)** CD4^+^ T cells were sorted by magnetic beads and stimulated. siRNA was then used to interfere with NFATc1. The expression of NFATc1 and PD-1 was detected by qPCR. 3–7 samples were prepared for each group, and the experiments were repeated three times. The error bars represented number of mice. *p < 0.05, **p < 0.01. ns, no statistical significance.

To further study the genes that initiate PD-1 expression in CD4 T cells in *P. yoelii* infected mice, this study compared the expression profiles of the reported 10 transcriptional activators/repressors (NFATc1, FoxO1, Notch, c-fos/AP-1, ISGF3, STAT3, NF- κB, T-bet, STAT4, Blimp-1) (20) using the sequencing data. As shown in **Figure 4A**, the expressions of NFATc1, Blimp-1, and T-bet between PD-1^+/-^ CD4 T cells were significantly different, and the FC of NFATc1 ranked No. 1. As mentioned above, Blimp-1 and T-bet were transcriptional repressors (20). Therefore, NFATc1 was speculated to be the key transcription factor to induce PD-1 expression after *P. yoelii* infection. FCM results showed that only PD-1^+^ CD4 T cells in the infected mice expressed NFATc1, and the other three groups of cells did not almost express NFATc1 (**Figure 4B**).

Subsequently, the dynamic changes of the expression of NFATc1 in CD4 T cells were monitored. The FCM results (**Figure 4C**) showed that its trend was similar to that of PD-1 in CD4 T cells (**Figure 1G**). The percentages of splenic NFATc1^+^ CD4 T cells were remarkably increased with prolonged infection and picked on day 16 (about 60%). Then the percentages gradually reduced (**Figure 4C**). These results indicated that NFATc1 could be the possible upstream regulating factor of PD-1 upon *P. yoelii* infection.

The distributions of NFATc1were explored. Western blot showed the PD-1^+^ CD4 T cells in the infected mice expressed more NFATc1, and NFATc1 was mainly distributed in the nucleus of PD-1^+^ CD4 T cells after *P. yoelii* infection (**Figure 4D**). In the ChIP assay, the efficiency of NFATc1 binding with PD-1 promoter in the *P. yoelii* infected mice was significantly higher than that in the naive mice (*p* < 0.01) (**Figure 4E**). These results suggested that NFATc1 is directly bound to the promoter sequences of PD-1 after *P. yoelii* infection. Then CD4 T cells were incubated with antibodies (anti-CD3 and anti-CD28). Both the expression of NFATc1 and PD-1 was up-regulated after the stimulation (**Figure 4F**). Subsequently, siRNA was used to interfere with NFATc1. Both the expression of NFATc1 and PD-1 was down-regulated (**Figure 4F**). Altogether, these results suggested that NFATc1 was a key transcription factor to induce PD-1 expression after *P. yoelii* infection.

### NFATc1 and PD-1 expression was induced through HIF-1α after *P. yoelii* infection

**Figure 5:**
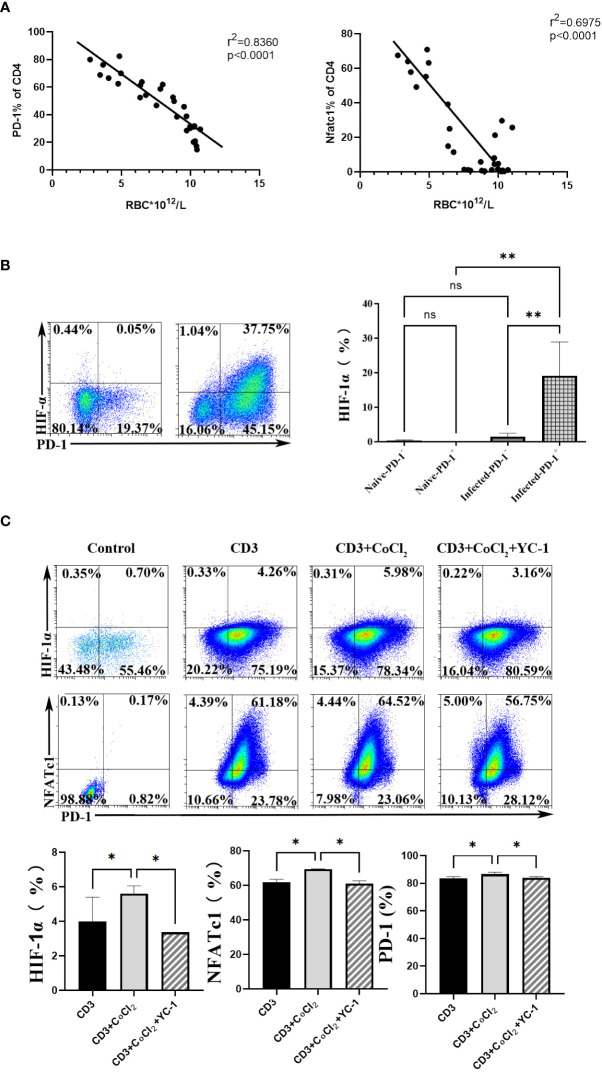
HIF-1α was an important factor for NFATc1 expression after *P. yoelii* infection. **(A)** the correlation ship between the expression of PD-1 and NFATc1 and the erythrocyte density in *P. yoelii* infected mice. **(B)** the expression of HIF-1α in PD-1^+/-^ CD4 T cells was verified by FCM. **(C)** 400 μM CoCl_2_ was used to induce hypoxic conditions for CD4 T cells. And then the specific inhibitor of HIF-1α (YC-1) was used. The expression of HIF-1α, NFATc1 and PD-1 was detected by qPCR. 3–7 samples were prepared for each group, and the experiments were repeated three times. The error bars represented number of mice. *p < 0.05, **p < 0.01. ns, no statistical significance.

To further explore the upstream genes which regulated NFATc1 in CD4 T cells upon *P. yoelii* infection, the erythrocyte density of the infected mice was tested and the correlation between the proteins and the erythrocyte density was investigated. The expression of PD-1 and NFATc1 in CD4 T cells were negatively correlated with the density of red blood cells in the mice (**Figure 5A**). The red blood cells play important roles in transporting oxygen. Anemia is one of the common symptoms of acute malaria. So, it was supposed that the expression of PD-1 and NFATc1 was related to hypoxia. HIF-1α plays a vital role in cell response to hypoxia (24). HIF-1α was highly expressed in PD-1^+^ cells in mRNA from the transcriptome sequencing data (**Table S1**). FCM results showed that only PD-1^+^ CD4 T cells in the infected mice expressed HIF-1α, while, HIF-1α could hardly be detected in the other three groups of cells (**Figure 5B**). Next, 400 μM CoCl_2_ (an inducer of HIF-1α) was used to induce hypoxic conditions for the sorted CD4 T cells. All expressions of HIF-1α, NFATc1, and PD-1 were up-regulated by CoCl_2_ stimulation. When the sorted CD4 T cells were treated with 400 μM CoCl_2_ and 1 μM YC-1 (a specific inhibitor of HIF-1α), expressions of HIF-1α, NFATc1, and PD-1 were down-regulated compared with that of cells treated with 400 μM CoCl_2_ only (**Figure 5C**).

**Figure 6:**
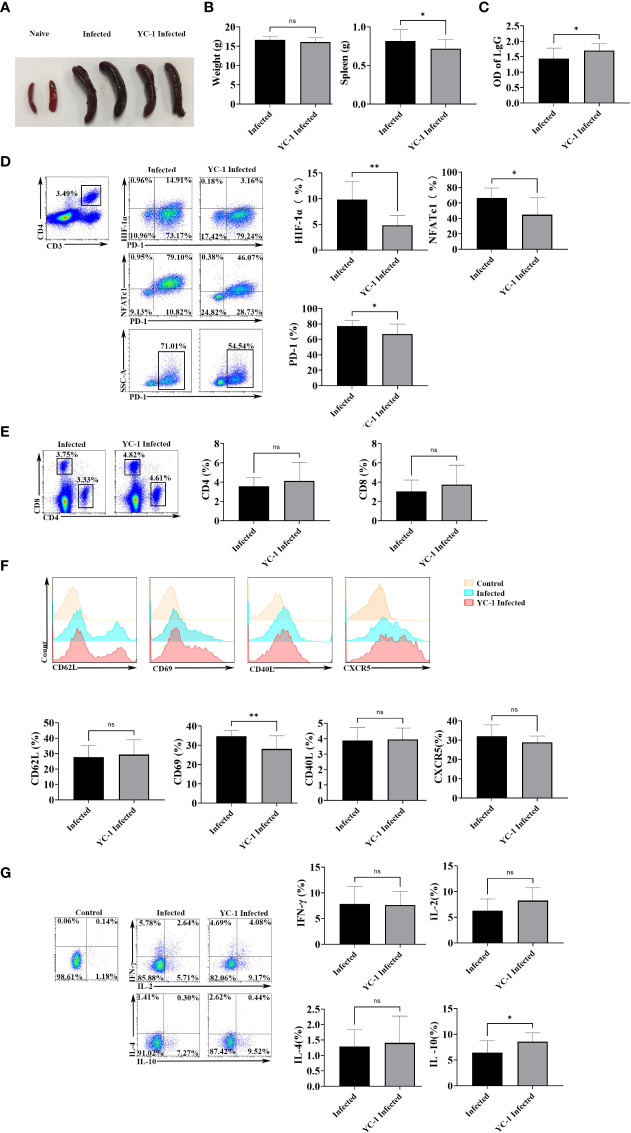
HIF-1α inhibitors possessed the ability to reduce the expression of HIF-1α, NFATc1, and PD-1. The mice were infected with *P. yoelii*, and then they were injected with YC-1 every day. The infected mice were set as controls. 12 days post-infection, the spleens were picked out. **(A)** the appearance of the spleens in the YC-1 infected and the infected groups. **(B)** the weight and the splenic weight of the mice were compared. **(C)** the IgG from the serum of the mice was compared. **(D)** the expressions of HIF-1α, NFATc1, and PD-1 in CD4 T cells in the infected and YC-1 infected mice were tested by FCM. **(E)**, the percentages CD4 T cells and CD8 T cells from the infected and YC-1 infected mice were compared.**(F)** the expressions of CD62L, CD69, CD40L, and CXCR5 in CD4 T cells from the infected and YC-1 infected mice were compared. **(G)** the percentages of IL-10, IFN-γ, IL-2, or IL-4 secreting CD4 T cells from the infected and YC-1 infected mice were compared. 3–7 samples were prepared for each group, and the experiments were repeated three times. The error bars represented number of mice. *p < 0.05, **p < 0.01. ns, no statistical significance.

Subsequently, mice were infected with *P. yoelii*, and then they were injected with YC-1 (10 mg/kg) every day. The naive mice and the infected mice were set as controls. Twelve days later, the spleens were removed. As shown in **Figures 6A, B**, spleens from the YC-1 infected mice were significantly smaller than those in the infected mice. However, the body weight of the two groups was similar. Moreover, IgG from the serum of the mice was tested by ELISA. Results (**Figure 6C**) indicated that a higher level of IgG was induced in the YC-1 infected mice. Then the splenic lymphocytes from the infected and YC-1 infected mice were isolated, and expressions of HIF-1α, NFATc1, and PD-1 in CD4 T cells were detected by FCM, respectively. Compared with the infected group, expressions of HIF-1α, NFATc1, and PD-1 were down-regulated in the YC-1 infected mice (**Figure 6D**). These results further indicated that NFATc1 and PD-1 expression could be induced in CD4 T cells through HIF-1α after *P. yoelii* infection.

The phenotype and cytokine expression from the infected and YC-1 infected mice were detected. As shown in **Figure 6E**, the percentages CD4 T cells and CD8 T cells were similar in the two groups (*p* > 0.05). Fewer CD4 T cells expressed CD69 in YC-1 infected mice comparing with that in the infected mice (*p* < 0.01), while, expressions of CD62L, CD40L, and CXCR5 were similar between the two groups of mice (*p* > 0.05) (**Figure 6F**). The proportion of IL-10 secreting CD4 T cells significantly increased in YC-1 infected mice (*p* < 0.05), but the percentages of IFN-γ, IL-2, and IL-4 secreting CD4 T cells were similar in the two groups (*p* > 0.05) (**Figure 6G**). The results further confirmed that PD-1^+^ CD4 T cells were more activated and could secrete more cytokines to modulate the host’s immune responses.

## Discussion

CD4 T cells are the central immune cells against *P. yoelii* infection (3, 4). PD-1 is a well-known key co-inhibitory factor (25). In this study, CD4 T cells expressed more PD-1 upon *P. yoelii* infection (**Figure 1**). CD62L and CD69 are the classic markers of T cell activation-associated molecules (26, 27). CD40L (28) and CXCR5 (29) are important molecules for CD4 T cells to facilitate the germinal center formation, B cells, and plasma cells differentiation. Compared with PD-1^-^ CD4 T cells, PD-1^+^ CD4 T cells expressed less CD62L and more CD69, CD40L, and CXCR5 (**Figure 2A**). Microarray results also found that PD-1^+^ CD4 T cells expressed less CD62L and more CXCR5 (**Figure 3E**). The results suggested that PD-1^+^ CD4 T cells would be strongly able to activate host immunity. Similarly, in mycobacterium tuberculosis (M.tb) individuals, PD-1^+^ CD4 T cells were not exhausted but showed greater activation (19).

Secreting cytokines is one important ability of CD4 T cells. IL-4 could promote B cells differentiation and maturation (30–32). IFN- γ plays a vital role in the host’s protective immunity against *Plasmodium* (6–8), which could synergistically promote the expression of nitric oxide synthase to control the load of parasites in the host (4, 9, 10). IL-2 is necessary for T cell proliferation and effector and memory cells differentiation (33). IL-10, a vital regulatory cytokine, could modulate the host’s immune responses against pathogens and thereby prevent severe damage to the host (34). In the present study, PD-1^+^ CD4 T cells could secrete more IFN-γ, IL-10, IL-4, and IL-2. The results that the spleens in the YC-1 infected group were significantly smaller (*p* < 0.05) further indicated that PD-1 was an anti-inflammatory molecule. Taken together, PD-1^+^ CD4 T cells were not exhausted but were more activated after *P. yoelii* infection. Similarly, the related studies also showed that PD-1 was a specific marker of Tfh cells, and Tfh cells were not exhausted T cells but were the core of maintaining antigen-specific effects (17–19). It was also reported that T cells expressing PD-1 molecule in acute malaria were not exhausted cells (35).

The microarray results of PD-1^+/-^ CD4 T cells could not only be used to analyze the role of PD-1 but also was useful to explore the upstream regulating molecules. Bioinformatic analyses indicated that the differentially expressed genes have been involved in immune response and the proliferation and differentiation of lymphocytes, especially B cells (**Figure 3**). PD-1 is a distinguishing factor of Tfh cells, which are necessary for maintaining the germinal center and regulating the differentiation and development of B cells (36).

PD-1 in CD4 T cells increased upon *P. yoelii* infection, and the upstream regulation molecules were not well-known. It was reported that PD-1 could be regulated by NFATc1, Notch, FoxO1, c-fos/AP-1, STAT3, NF- κB, ISGF3, Blimp-1, STAT4, T-bet (20). It was also reported that NFATc1 was required to initiate PD-1 expression, and NFATc1 specific inhibitors could restrain the expression of PD-1 in T cells (37, 38). The difference was statistically significant only in one transcriptional activator (NFATc1) between PD-1^+/-^ CD4 T cells sorted from mice infected with *P. yoelii* (**Figure 4A**). Further experiments also confirmed that NFATc1 was the key transcriptional factor for CD4 T cells after *P. yoelii* infection (**Figure 4**).

Anemia is one of the common symptoms of acute malaria. It is well known that red blood cells play important roles in transporting oxygen. It would be a hypoxic environment for the body, and many molecules would be regulated in acute malaria. Expressions of PD-1 and NFATc1 in CD4 T cells were significantly negatively correlated with the density of red blood cells in mice (**Figure 5A**). HIF-1α plays a vital role in cell response to hypoxia (24). It was reported that more HIF-1α and NFATc1 were expressed in hypoxic conditions (39, 40). NFATc1 activation was dependent on HIF, and NFATc1 was upregulated in hypoxia (41). In the present study, HIF-1α was highly expressed in PD-1^+^ CD4 T cells after *P. yoelii* infection (**Figure 5**). Both the inducer and the inhibitor of HIF-1α could significantly change the expression of NFATc1 and PD-1 *in vitro*. *In vivo*, when *P. yoelii*-infected mice were treated with HIF-1α inhibitor (YC-1), expressions of HIF-1α, NFATc1, and PD-1 were down-regulated (**Figure 5**). This study illustrated that HIF-1α was the upstream molecular of NFATc1 and PD-1 during *P. yoelii* infection. PD-1 ligand 1 (PD-L1) was reported to be a novel direct target of HIF-1α (22). This study was the first report that HIF-1α was one upstream molecule of PD-1.

In summary, this study showed that *P. yoelii* infection could induce NFATc1 expression through HIF-1 α. The highly expressed NFATc1 entered the nucleus of CD4 T cells and initiated PD-1 expression in CD4 T cells. PD-1^+^ CD4 T cells appeared to be more activated and could secrete more cytokines to modulate host immune responses.

## Data availability statement

The datasets presented in this study can be found in online repositories. The names of the repository/repositories and accession number(s) can be found below: Gene Expression Omnibus (GEO) database with accession number GSE199687.

## Ethics statement

The animal study was approved by the Institutional Animal Care and Use Committee of Guangzhou Medical University (S2020-055).

## Author contributions

HW, AX, JL, and CF performed the *in vivo* and *in vitro* cellular tests. LL, JX, and FS prepared parasites and animals. FM, DC, HX, and QY analyzed the results. XP and JH contributed to the writing of the paper. XT and JH conceived the study. All authors read and approved the final manuscript.

## Funding

This research was supported by grants from the Natural Science Foundation of Guangdong province (2020A1515010251, 2021A1515011032), Guangdong Basic and Applied Basic Research Foundation (2021A1515220034), Guangzhou science and technology project (202002030082, 202102020912), Key Discipline of Guangzhou Education Bureau (Basic Medicine) (201851839) and the Open Foundation Key Laboratory of Tropical Diseases Control (Sun Yat-sen University), Ministry of Education (2021kfkt03).

## Conflict of interest

The authors declare that the research was conducted in the absence of any commercial or financial relationships that could be construed as a potential conflict of interest.

## Publisher’s note

All claims expressed in this article are solely those of the authors and do not necessarily represent those of their affiliated organizations, or those of the publisher, the editors and the reviewers. Any product that may be evaluated in this article, or claim that may be made by its manufacturer, is not guaranteed or endorsed by the publisher.

## Supplementary material

Supplementary Table 1 The information for the differentially expressed genes in microarray transcriptome sequencing data.

Supplementary Figure 1 The gating strategy used for all FACS data.
