# Supplementary figures and images for: PD-1+ CD4 T cell immune response is mediated by HIF-1α/NFATc1 pathway after P. yoelii infection
Source: Front Immunol. 2022 Aug 24;13:942862. doi: 10.3389/fimmu.2022.942862 (PMC9449323; doi:10.3389/fimmu.2022.942862)

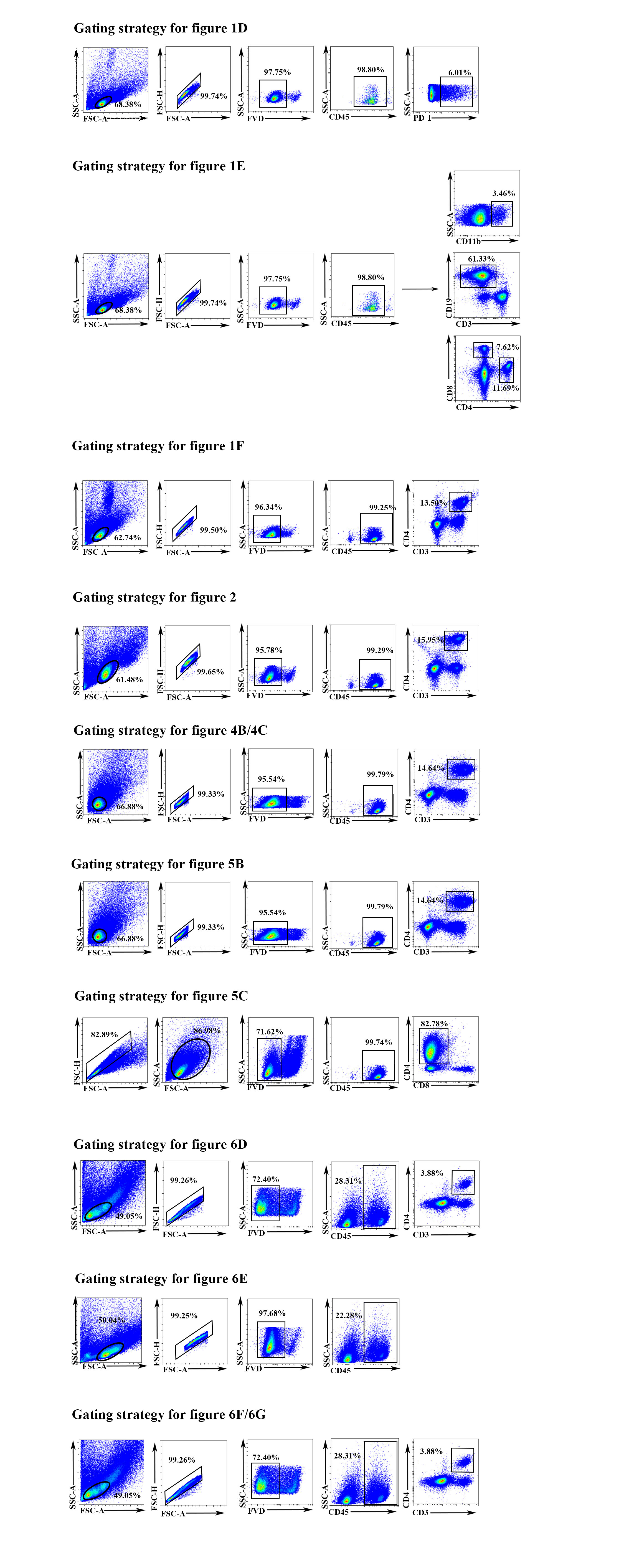

Supplement: Supplementary Figure 1 — The gating strategy used for all FACS data. [file Image_1.jpg]
